# Efficacy and Safety of Tranexamic Acid in Intertrochanteric Fractures: A Single‐Blind Randomized Controlled Trial

**DOI:** 10.1111/os.12511

**Published:** 2019-08-16

**Authors:** Xin‐die Zhou, Yi Zhang, Li‐feng Jiang, Jun‐jie Zhang, Dong Zhou, Li‐dong Wu, Yong Huang, Nan‐wei Xu

**Affiliations:** ^1^ Department of Orthopaedics The Affiliated Changzhou No. 2 People's Hospital of Nanjing Medical University Changzhou China; ^2^ Department of Orthopaedic Surgery The Second Affiliated Hospital, Zhejiang University School of Medicine Hangzhou China

**Keywords:** Blood loss, Intertrochanteric fracture, Randomized controlled trial, Tranexamic acid, Transfusion

## Abstract

**Objective:**

To investigate the efficacy and safety of tranexamic acid (TXA) in the reduction of bleeding and the need for transfusion in elderly intertrochanteric fracture patients.

**Methods:**

A total of 100 patients with intertrochanteric fractures undergoing surgery were enrolled and randomly allocated to the TXA group in which patients (75.10 ± 8.27 years old) were treated with 1 g of TXA, or the control group (77.82 ± 6.42 years old) treated with a placebo. Surgery was performed by two senior orthopaedic surgeons from two institutions. The proximal femoral nail antirotation (PFNA) was conducted using the standard procedure. Three outcome measures, including blood loss, transfusion, and complications, were recorded. Blood loss and transfusion were investigated to assess TXA's effectiveness, while complications were investigated to assess TXA's safety. Statistical indicators for blood loss included total, intraoperative, postoperative, and hidden blood loss volumes, calculated by hemoglobin levels, hematocrit levels, and drainage volume. The number and amount of blood transfusions were recorded. Complications associated with surgery, including deep vein thrombosis, pulmonary embolism, wound hematoma, wound infection, cardiovascular and cerebrovascular accidents, and respiratory infections, were also recorded.

**Results:**

All patients were followed up for 1 month after surgery. There were no significant differences in demographic and clinical characteristics between the two groups. The TXA group suffered significantly less total blood loss (563.37 ± 197.51 *vs* 819.25 ± 273.96 mL, 95% *CI*: −349.49 to −162.27, *P* < 0.01), intraoperative blood loss (140.3 ± 80.64 *vs* 230.5 ± 130.56 mL, 95% *CI* −132.74 to −47.66, *P* < 0.01), and hidden blood loss (410.42 ± 178.23 *vs* 571.19 ± 218.13 mL, 95% *CI*: −238.85 to −82.69, *P* < 0.01) than the control group. However, postoperative total blood loss was not significantly different (97.5 ± 20.93 *vs* 94.7 ± 35.78 mL; *P* = 0.63). A total of 5 patients from the TXA group and 27 from the control group received packed RBC for postoperative transfusion, but the mean number of transfusion units was not significantly different between groups. Complications including deep vein thrombosis, pulmonary embolism, myocardial infarction, ischemic cerebral infarction, hematoma, and infection were observed in both groups, but no significant differences were found.

**Conclusions:**

In intertrochanteric fracture surgery performed using PFNA, intravenous administration of TXA significantly reduced the risk of intraoperative, total and hidden blood loss, in addition to the need for allogeneic transfusion, without increasing the rate of complications.

## Introduction

Compounded by the problems of aging, hip fractures are a common injury in the elderly. Their annual incidence is increasing rapidly and is projected to surpass 6.3 million by 2050^1^, ^2^. Hip fractures are arguably the most important public health problem faced by orthopaedic surgeons. More than 250 000 hip fractures occur annually and mostly in the elderly, with the 1‐year mortality rates ranging from 14% to 36%, due to the frequent association with osteoporosis^3^, ^4^. Their treatment consumes a growing percentage of healthcare expenditure. Hip fractures are anatomically classified in relation to the hip capsule as intracapsular (i.e. at the femoral neck) or extracapsular (i.e. intertrochanteric or subtrochanteric fractures). Intertrochanteric fractures (ITF) and femoral neck fractures represent the majority of hip fractures, occurring with similar frequency. Fractures below the femoral neck are referred to as intertrochanteric fractures, and those below the lesser trochanter as subtrochanteric fractures. Patients who have had an intertrochanteric fracture are at risk of cardiovascular or pulmonary complications and problems associated with infections, bleeding, and thrombosis, possibly resulting in death. Due to preoperative levels of patient activity and their dependence on a daily routine, nearly 30% of intertrochanteric fracture patients die within the first 12 months; elderly patients are more likely to die due to their relative inactivity^5^, ^6^. The goals of care are to restore function while attaining the lowest possible rate of surgical and medical complications. Achieving stable reduction and fixation of the fracture, thereby permitting immediate mobilization, is key to these goals. Functional outcomes and mortality are associated with several factors, especially perioperative anemia and operative blood loss^7^, ^8^. To prevent or reduce blood loss during the perioperative period, it has been established that minimal invasive surgical therapy significantly reduces trauma with reliable efficacy and has, thus, been popularized. However, overall blood loss volume may be much larger than that reported. Median total blood loss in patients with extra‐capsular fracture of the hip (AO types 31‐A2.2 to 31‐A3) treated with short intramedullary nails has been reported to be 2100 mL^9^. ITF patients often receive red blood cell transfusions to correct anemia resulting from blood loss due to the fracture or surgery. However, blood transfusions are not without risk. Researchers have proposed other methods, such as permissive hypotension, topical freezing saline, thromboplastic agents, auto‐transfusion devices, and administration of erythropoietin, autologous blood transfusion or anti‐fibrinolytic agents^10^, ^11^, ^12^. Despite their effectiveness, these techniques may still be associated with multiple shortcomings, such as the required environment during deployment, economic factors, risk of thrombosis, heart and brain disorders, and the limitations imposed by the duration for which they are effective.

Tranexamic acid (TXA), a competitive inhibitor of tissue plasminogen activator that blocks the lysine‐binding sites of plasminogen^13^, has been widely used for the reduction of bleeding after trauma and during surgery^14^. A large trial involving 20 211 adult trauma patients reported that early administration of TXA was safe and effectively reduced the risk of death in those patients that were bleeding^15^. Because these benefits have been demonstrated in several clinical trials^16^, ^17^, TXA has recently provoked interest in orthopaedic surgery. Wide incorporation of TXA into everyday clinical practice is now promoted following the reporting of a number of significant investigations into its use in joint replacement and spine surgery^14^, ^18^. Clinical trials have established that TXA is effective in reducing blood loss in spine surgery without incremental risk or complications^19^, ^20^. Large prospective studies and meta‐analyses have demonstrated the effectiveness and safety of TXA in total knee and hip arthroplasty^14^, ^21^. Despite the extensive study of TXA in spine and arthroplasty surgery, a paucity of studies regarding its use in orthopaedic trauma surgery has limited its integration into the field, although it is these patients that may benefit most from TXA therapy. Recently, a number of studies^9^, ^22^, ^23^, ^24^, ^25^, ^26^, ^27^ have reported on the use of TXA in ITF surgery, but due to varying methods of administration and dosage, no consistent conclusions can be drawn.

Therefore, we performed a randomized controlled trial (RCT) to investigate the efficacy and safety of the intravenous administration of 1 g TXA in elderly ITF patients undergoing surgery using the proximal femoral nail antirotation (PFNA) system, exploring in particular the following three points: (i) whether TXA is able to reduce blood loss; (ii) whether the use of TXA reduces the proportion of surgery requiring a transfusion; and (iii) whether the use of TXA increases the probability of complications.

## Materials and Methods

### *Inclusion and Exclusion Criteria for Study Population*

In this study, a prospective multi‐center single‐blinded RCT, patients with stable or unstable ITF admitted to two hospitals between 1 July 2017 and 30 April 2018 were eligible to be included. The study was approved by the Institutional Ethics Committees of the Affiliated Changzhou No. 2 People's Hospital of Nanjing Medical University and the Second Affiliated Hospital of Medical College, Zhejiang University, and was performed in line with international ethical guidelines for studies involving human subjects (the Declaration of Helsinki)^28^. This study was also conducted and reported in accordance with the Consolidated Standards of Reporting Trials (CONSORT) statement^29^. Inclusion criteria were as follows: (i) a definite history of trauma and a confirmed diagnosis of ITF classified according to AO type by X‐ray or CT; (ii) presence of pain, dysfunction, and local swelling of the hip with limited function in the injured limb; and (iii) eligibility for intertrochanteric fracture surgery using the PFNA system, as determined by the senior orthopaedic surgeon. Exclusion criteria were as follows: (i) allergy to TXA; (ii) recent or ongoing thromboembolic events (including deep venous thrombosis, pulmonary embolism, arterial thrombosis, cerebral thrombosis, or stroke); (iii) recent anticoagulation or hemostatic therapy; (iv) having a condition leading to the impairment of coagulation function; (v) history of other diseases (malignancy, preoperative hepatic or renal dysfunction, diabetes, injury of other organs, prior surgery on the injured hip, and serious cardiac, respiratory or other chronic disease) that may influence outcome; and (vi) pathological fracture. Data collected included gender, age, height, weight, fracture type (Orthopedic Trauma Association [AO/OTA] classification) and general preoperative condition according to the American Society of Anesthesiologists (ASA) classification. Written informed consent was obtained from all patients before inclusion and randomization.

### *Randomization and Operative and Perioperative Procedures*

Patients were randomized using a random number table into either a TXA or control group, and were either infused intravenously 15 minutes prior to surgery with TXA (1 g/100 mL) or received nothing, respectively. Patients and the two authors who pooled the data were blinded to the study procedure, but the allocation was not concealed from the surgeons. The surgical procedure was standardized, under spinal anesthesia in the horizontal position within 72 h of admission. Surgery was performed by two senior orthopaedic surgeons with 10 years of experience from the two institutions. Fractured bone fragments were identified by X‐ray with the patients in a supine position, and PFNA was then performed using the standard procedure, with a low‐vacuum drain inserted into the sub‐muscular plane. All patients received one preoperative dose and two postoperative doses of intravenous second‐generation cephalosporin. All patients received standard thromboprophylaxis with low‐molecular‐weight heparin from the second day after admission to 24 h prior to surgery, and for 12 h after surgery. After discharge, patients were followed up after the first month. The indication for blood transfusion was set as <70 g/L hemoglobin. The hemoglobin and hematocrit levels were measured 1 day before and the first and third day following surgery, with visible blood loss collected using a suction apparatus and gauze measured during the procedure. The duration of surgery and postoperative drain outputs were recorded.

### *Outcome Measures of Blood Loss*

The primary outcome was total blood loss as calculated from the difference between the preoperative hematocrit and the lowest postoperative hematocrit during the hospital stay or the lowest postoperative hematocrit prior to blood transfusion. The postoperative hematocrit value, used in the calculation of blood loss, was the smaller volume of that collected on the first and third postoperative day. Estimated blood loss was evaluated using the Gross equation and Nadler's formula^30^, ^31^, ^32^:

$$\text{Women blood volume}\;\left(L\right)=\text{Height}\;{\left(m\right)}^{3}\times 0.356+\text{Weight}\;\left(\mathrm{kg}\right)\times 0.033+0.183$$

$$\mathrm{Men}\;\text{blood volume}\;\left(L\right)=\text{Height}\;{\left(m\right)}^{3}\times 0.356+\text{Weight}\;\left(\mathrm{kg}\right)\times 0.032+0.604$$

$$\text{Estimated total blood loss}\;\left(L\right)=\text{Blood volume}\times \left({\mathrm{Hct}}_{\text{preop}}-{\mathrm{Hct}}_{\text{postop}}\right)/\left(\left({\mathrm{Hct}}_{\text{preop}}+{\mathrm{Hct}}_{\text{postop}}\right)/2\right).$$

Intraoperative blood loss was recorded using a suction apparatus and gauze, measured during the procedure. Postoperative blood loss was recorded using postoperative drain outputs.

Hidden blood loss usually includes blood extravasating into the tissues, remaining in the joint cavities, and lost *via* hemolysis. It is considered to be the main reason why the postoperative hemoglobin is lower than anticipated in hip surgery patients^14^. The formulae used were as follows:

$$\text{Visible blood loss}=\left(\text{Intraoperative blood loss}+\text{Postoperative blood loss}\right)\times \left({\mathrm{Hct}}_{\text{preop}}+{\mathrm{Hct}}_{\text{postop}}\right)/2$$

Hidden blood loss=Estimated total blood loss−Visible blood loss+Transfusion blood.

### *Outcome Measures of Transfusion*

Transfusion remains another major problem, because of considerable blood loss in ITF surgery. Such transfusion of allogeneic erythrocytes is not free of adverse events and has been associated with transmission of infectious diseases, bacterial infection, immune sensitization, intravascular hemolysis, transfusion‐induced coagulopathy, renal failure, and even death^14^. Patients were transfused if postoperative hemoglobin level was <70 g/L, and we recorded the volume of transfused blood.

### *Outcome Measures of Complications*

Venous thromboembolism, whose main manifestations are deep vein thrombosis and pulmonary embolism, was the major recorded complication. It is a major global burden, along with substantial morbidity and mortality. No radiological investigation was performed to assess thrombosis unless indicated clinically. Deep vein thrombosis and pulmonary embolism were confirmed by compression ultrasonography and spiral CT, respectively. Other non‐specific complications, or complications associated with surgery, including wound hematoma, wound infection, cardiovascular or cerebrovascular accidents, and respiratory infections, were also recorded.

### *Statistical Analysis*

Two authors independently pooled data from each case then conducted statistical analysis using SPSS 18.0 (SPSS, Chicago, IL, USA). Descriptive data are presented as mean ± standard deviation (*SD*). Odds ratio (*OR*) with 95% confidence intervals (*CI*) or mean differences (*MD*) with 95% *CI* were calculated for dichotomous and continuous outcomes, respectively. Numerical and measured data were compared using a *t*‐test and χ^2^‐test, respectively. An appropriate power and sample size calculator (version 1.2, HyLown Consulting LLC, Atlanta, USA) was used to calculate statistical power. The following parameters were used in the calculations: α, probability of a type I error for a two‐sided test; P0, probability of exposure in controls; *N*, number of patients; m: ratio of control to experimental subjects; and Ψ, odds ratio of exposure in experimental subjects relative to controls. *P* < 0.05 was considered significant.

## Results

### *Characteristics of Participant Flow and Follow‐up*

**Figure 1:**
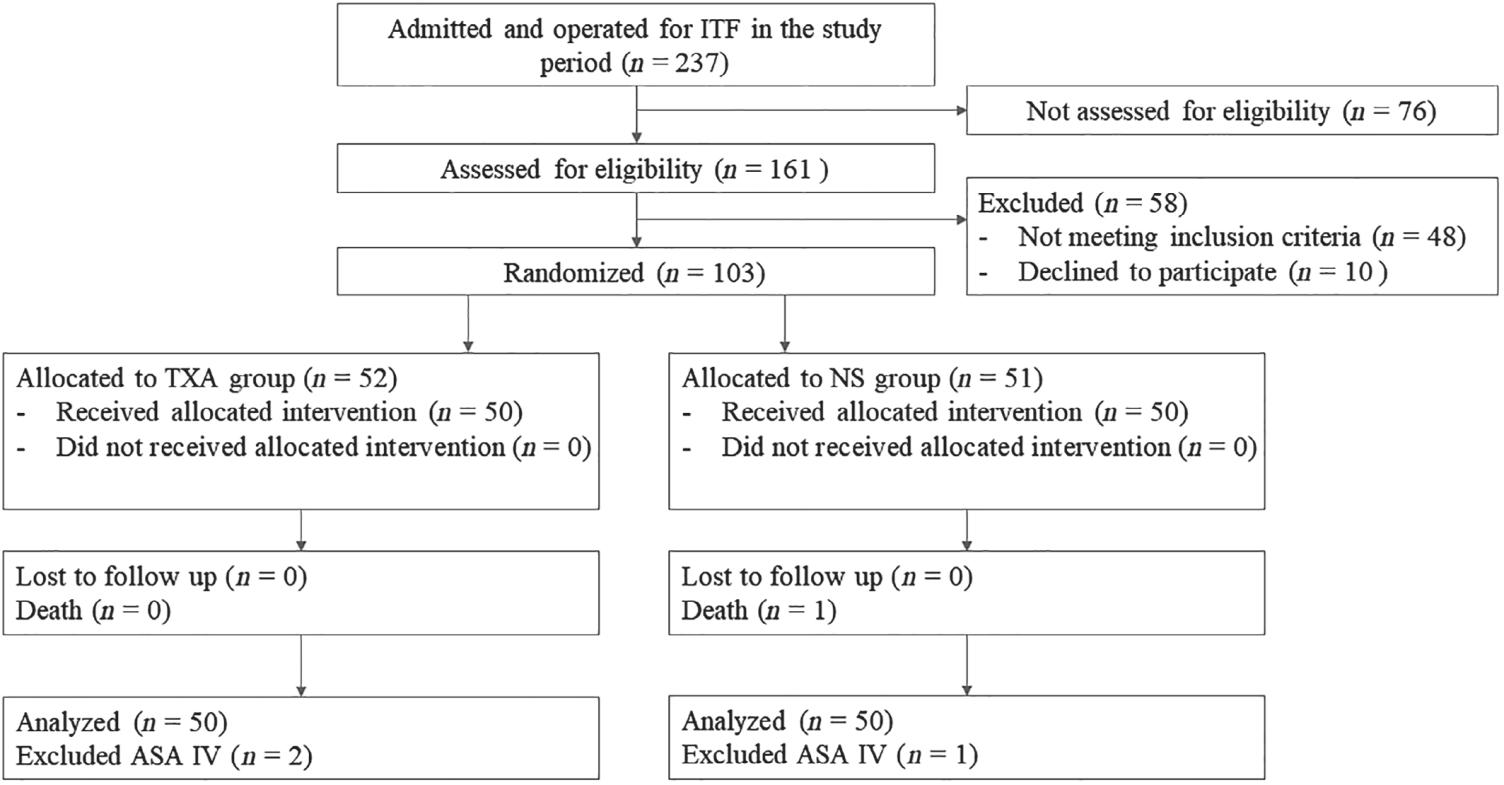
Flow chart of patient enrollment and Consolidated Standards of Reporting Trials (CONSORT).

**Figure 2:**
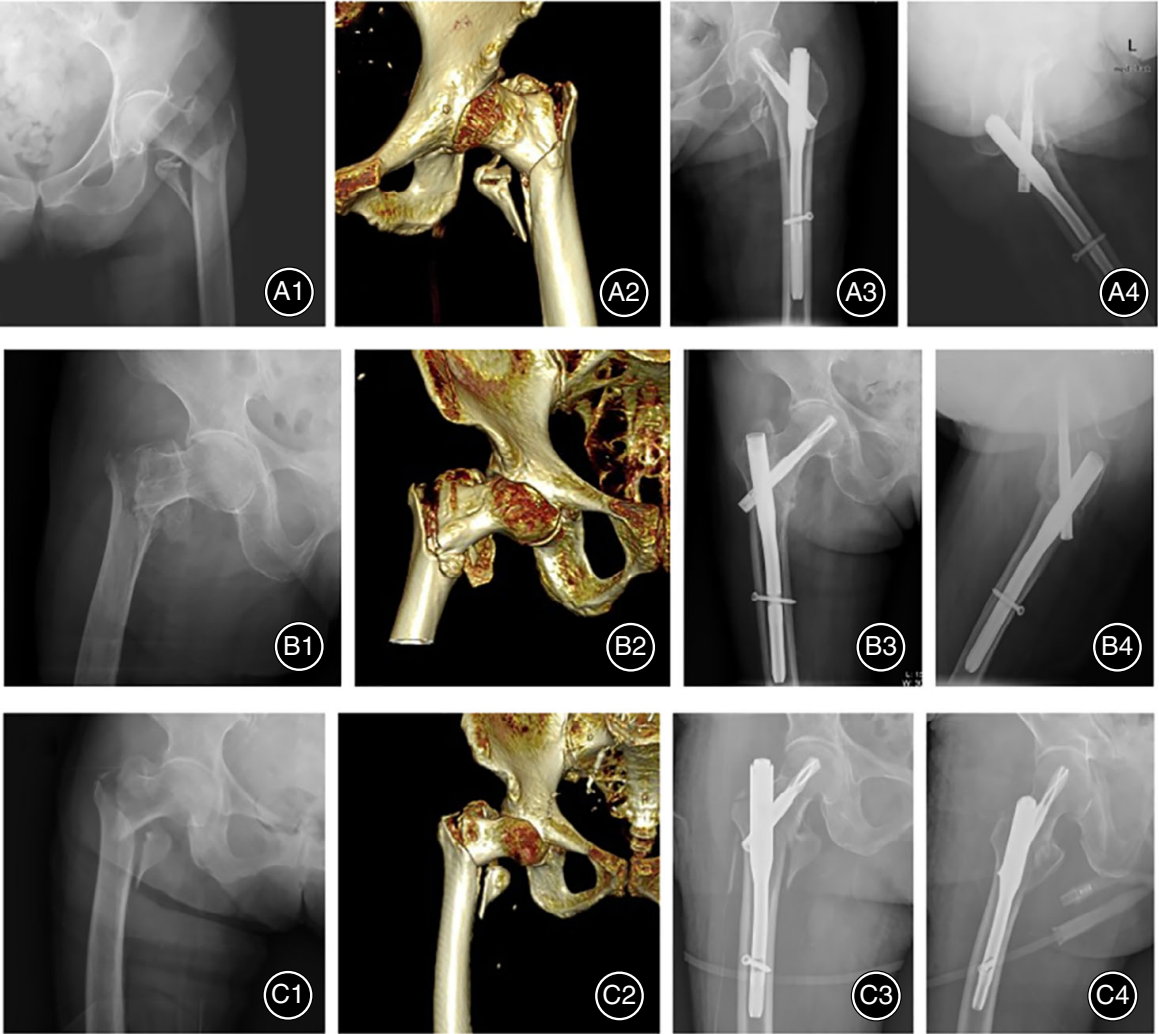
Preoperative X‐rays (A1, B1, C1) and CT scans (A2, B2, C2) of femoral intertrochanteric fracture from three patients ((A) 77‐year‐old female; (B) 84‐year‐old female; (C) 78‐year‐old male), and which had been treated by closed reduction and internal fixation with PFNA (A3, A4, B3, B4, C3, C4).

Between July 2017 and April 2018, 237 ITF patients were admitted to the two institutions through their respective emergency departments. One hundred patients who met all inclusion criteria and were not excluded by virtue of any exclusion criteria were randomized into a TXA (*n* = 50) or control group (*n* = 50) (Fig. 1). All surgical procedures were successful and the patients were followed up for 1 month postoperatively (Fig. 2). There were no significant differences in age, body mass index (BMI), preoperative hemoglobin levels, preoperative hematocrit level, duration of surgery or hospital stay, or demographic or clinical characteristics between groups (Table 1). Four patients in the TXA group received a total of 7.5 U packed red blood cells (RBC) by intravenous infusion prior to surgery, compared with 3 patients in the control group who received 6 U.

**Table 1:** Demographic and clinical characteristics of the included patients

Variables	TXA group (*n* = 50)	Control group (*n* = 50)	*P‐*value
Female (%)	70	56	
Age (Mean [*SD*])	75.10 (8.27)	77.82 (6.42)	0.07
BMI (Mean [*SD*])	21.93 (6.58)	22.85 (5.35)	0.44
Side (right, %)	60	62	
Preop.hemoglobin level (g/L)	101.60 (20.18)	107.87 (18.72)	0.11
Preop.hematocrit level (%)	32.54 (3.64)	31.59 (3.99)	0.22
Preop.transfusion (*n*)	4	3	
Preop.transfusion (U)	7.5	6	
AO fracture classification (*n*)			
31 A1	27	25	
31 A2	17	20	
31 A3	6	5	
ASA classification (*n*)			
I	5	6	
II	32	35	
III	11	8	
IV	2	1	
Operative time (min, mean [*SD*])	75.53 (24.86)	79.88 (21.51)	0.35
Hospital stay (days, Mean [*SD*])	10.71 (2.41)	11.05 (3.15)	0.54

TXA, tranexamic acid.

### *Outcome Measures of Blood Loss*

The TXA group suffered significantly less total blood loss (563.37 ± 197.51 *vs* 819.25 ± 273.96 mL, 95% *CI*: −349.49 to −162.27, *P* < 0.01), intraoperative blood loss (140.3 ± 80.64 *vs* 230.5 ± 130.56 mL, 95% *CI* −132.74 to −47.66, *P* < 0.01), and hidden blood loss (410.42 ± 178.23 *vs* 571.19 ± 218.13 mL, 95% *CI*: −238.85 to −82.69, *P* < 0.01) than the control group. On postoperative day 1, the use of TXA significantly ameliorated the postoperative hemoglobin decrease, by 12.7 g/L in total (95% *CI*, 3.51 to 21.89 g/L; *P* < 0.01) and the hematocrit decrease, by 4.35% in total (95% *CI*, 2.13 to 6.57%; *P* < 0.01), but postoperative total blood loss was not significantly different (97.5 ± 20.93 *vs* 94.7 ± 35.78 mL; *P* = 0.63; Table 2).

**Table 2:** Comparison of postoperative clinical outcomes between the TXA group and NS group

Variables	TXA group (*n* = 50)	Control group (*n* = 50)	*MD*/*OR*	95% confidence interval	*P‐*value
Intraoperative blood loss (mL, mean [*SD*])	140.30 (80.64)	230.50 (130.56)	−90.20	−132.74 to − 47.66	<0.01
Postoperative day 2 drainage (mL, mean [*SD*])	97.50 (20.93)	94.70 (35.78)	2.80	−8.69 to 14.29	0.63
Hemoglobin postop. Day 1 (g/L, mean [*SD*])	92.51 (18.45)	79.81 (27.56)	12.70	3.51 to 21.89	<0.01
Hemoglobin postop. Day 3 (g/L, mean [*SD*])	86.79 (25.80)	80.48 (36.42)	6.31	−6.06 to 18.68	0.32
Hematocrit postop. Day 1 (%, mean [*SD*])	29.50 (6.23)	25.15 (5.02)	4.35	2.13 to 6.57	<0.01
Hematocrit postop. Day 3 (%, mean [*SD*])	28.85 (5.49)	27.06 (6.89)	1.79	−0.65 to 4.23	0.15
Transfusion rate (*n*, %)	5 (10%)	27 (54%)	0.09	0.03 to 0.28	<0.01
Transfusion units (U, mean [*SD*])	2.30 (0.97)	2.27 (0.76)	0.03	−0.31 to 0.37	0.86
Estimated total blood loss day 3 (mL, mean [*SD*])	563.37 (197.51)	819.25 (273.96)	−255.88	−349.49 to − 162.27	<0.01
Estimated hidden blood loss day 3 (mL, mean [*SD*])	410.42 (178.23)	571.19 (218.13)	−160.77	‐ 238.85 to ‐ 82.69	<0.01

TXA, tranexamic acid; MD, mean difference; OR, odds ratio.

### *Outcome Measures of Transfusion*

The number of patients who required packed RBC for postoperative transfusion was significantly (44%) less in the TXA group than the control group (95% *CI*, 0.03 to 0.28; *P* < 0.01), but the mean number of transfusion units was not significantly different between groups (Table 2). In addition, the power analysis indicated that the power of this study to detect the effect of TXA treatment on the transfusion rate was 99.9%, assuming an OR of 0.09.

### *Outcome Measures of Complications*

Two patients in the TXA group and three in the control group experienced deep vein thrombosis detected by doppler ultrasound prior to leaving hospital, a difference that was not significantly different (*P* = 0.65). However, one patient in the control group, who was diagnosed with DVT, died of pulmonary embolism (PE) on postoperative day 4. The other four patients were successfully treated using thrombolytic therapy. Other complications, including myocardial infarction, ischemic cerebral infarction, hematoma, and infection were also observed while in hospital, without any significant difference between groups (Table 3). All complications were treated satisfactorily, all patients having a favorable prognosis at the 1‐month follow up.

**Table 3:** Postoperative complications in the TXA group and control group

Complications	TXA group (*n* = 50)	Control group (*n* = 50)	*P*‐value
Medical			
Deep vein thrombosis	2	3	0.65
Pulmonary embolism	0	1	0.31
Myocardial infarction	0	1	0.31
Ischemic cerebral infarction	1	2	0.56
Surgical site			
Hematoma	1	1	0.46
Infection	0	1	0.31

TXA, tranexamic acid.

## Discussion

TXA is an antifibrinolytic agent that has been widely used to reduce bleeding following trauma and surgery, including cardiac surgery with and without cardiopulmonary bypass^33^, total hip and knee replacement^14^ and prostatectomy^34^. However, there are few studies (Table 4) investigating its safety and effectiveness in ITF surgery, with no consistent conclusion reached^35^. Thus, in this single‐blind RCT, it was found that the use of TXA significantly reduced total, intraoperative and hidden blood losses, in addition to reducing the number of patients who required allogeneic transfusions, without increasing the risk of thromboembolism.

**Table 4:** Characteristic of other related studies of tranexamic acid in ITF patients

Study and year	Country	Design	Sample size(cases/controls)	TXA group	Control group	DVT PPX	Anesthesia method	Surgical procedure	Drainage	Transfusion trigger	Follow up
Mohib_2015^23^	Pakistan	DB‐RCT	50/50	15mg/kg, IV, preoperation and postoperation	Normal saline	Enoxaparin	*NM*	*NM*	*NM*	< 7g/dl	Discharge
Baruah_2016^24^	India	RCT	30/30	15mg/kg, IV, preoperation	Normal saline	*NM*	Spinal anesthesia	DHS	Yes	<8.5 g/dl	Discharge
Drakos_2016^25^	Greece	DB‐RCT	100/100	3g, local administration, before closure	None	LMWH	Spinal anesthesia	Short cephalomedullary nail (GAMMA3)	None	<8 g/dl	12 months
Tengberg_2016^9^	Denmark	DB‐RCT	33/39	1g, IV, preoperation; 3g, IV, postoperation	Placebo	LMWH	Epidural analgesia	Short intramedullary nail (IMH)	*NM*	<9.96 g/dl	4 months
Virani_2016^26^	India	RCT	67/70	2g, local administration, before closure	None	*NM*	Spinal anesthesia	DHS and Barrel plate	Yes	*NM*	Discharge
Lei_2017^27^	China	RCT	37/40	1g, IV, preoperation	Normal saline	*NM*	*NM*	PFNA	Yes	< 9 g/dL	1 month
Tian_2018^22^	China	RCT	50/50	10 mg/kg, IV, preoperation and postoperation	None	LMWH	*NM*	Intramedullary nail	Yes	< 9 g/dL	4 months

DB, double blind; RCT, randomized controlled trial; IV, intravenous administration; NM, not mentioned; DHS, dynamic hip screw; LMWH, low molecular weight heparin; PFNA, proximal femoral nail antirotation

Tranexamic acid acts by binding to plasminogen and blocking the interaction of plasmin with fibrin, thereby preventing the fibrin clot dissolution^36^. For this reason, it has been established that it significantly reduces all causes of mortality and death due to bleeding in trauma patients suffering significant bleeding, particularly when administered early after injury^37^. It has been predicted that TXA use in surgery and following trauma would be cost‐effective and potentially life‐saving^38^. Thus far, the use of TXA in orthopaedic surgery has focused on arthroplasties and spinal surgery. In addition, the outcomes are partially positive: TXA can significantly reduce blood loss and blood transfusion requirements^14^, ^39^, ^40^. However, studies of TXA use in fracture surgery remain limited. Femoral ITF are one clinical group of common fractures, and are especially experienced by the elderly. The number of hip fractures has been projected to increase globally from 1.66 million in 1990 to 6.26 million in 2050^41^. Surgery is required for almost all ITF. Blood loss occurs as a consequence of both fracture and surgery and, thus, RBC transfusion is frequently used. However, blood transfusions are correlated with an increased risk of bacterial infections, possibly increased mortality, and the substantial costs involved in blood collection, preparation, transport, and administration^42^, ^43^. It has previously been reported that TXA reduces the requirement for erythrocyte transfusions but may promote a hypercoagulable state^44^. Moreover, its efficacy is inferior to that observed in hip or knee arthroplasty. Three other studies^9^, ^23^, ^25^ similarly confirm the effectiveness of TXA administration in elderly patients undergoing ITF surgery. Blood loss, transfused blood units, and healthcare costs were reported to be significantly reduced. Two other studies^22^, ^27^ in addition to the present trial have verified the effectiveness of TXA in the reduction of hidden blood loss.

Despite the effectiveness of TXA, an important, secondary research focus is its safety, especially thromboembolic events. In this regard, three questions that must be addressed are whether TXA influences the fibrinolytic system postoperatively, whether it also affects prothrombin times, activated partial thromboplastin times, international normalized ratios or platelet concentrations, and whether TXA interacts with thromboprophylaxis agents. The incidence of deep vein thrombosis (DVT) can be as high as 80% in ITF patients^45^. Therefore, investigating the efficacy and safety of TXA in ITF surgery is of great significance. The outcomes in this study are consistent with previous studies^9^, ^22^, ^27^, although a number of complications, including DVT, PE, myocardial infarction, ischemic cerebral infarction, hematoma, and infection, were observed. However, because no significant differences were observed between groups, with only small dosages being used during surgery, we were unable to ascertain with certainty the cause of these complications.

Despite the effectiveness and safety of TXA in ITF, there remain a number of difficulties that require addressing. The methods and dosages used in various studies are inconsistent. TXA was administered intravenously in six published studies^9^, ^22^, ^23^, ^24^, ^27^, with dosages ranging from 1 to 4 g in total. We found a maximal weighted mean difference of 570.8 mL compared with the largest dosage of 4 g, implying greater effectiveness in higher doses on the premise of safety. Drakos *et al*. and Virani *et al*.^25^, ^26^ report on local or intramuscular administration surrounding the wound, with only Drakos *et al*.^25^ demonstrating effectiveness in reducing blood loss and need for transfusion. It was concluded in these cases that TXA did not play a significant role in the reduction of postoperative blood loss or volume of blood transfusions when used locally in ITF surgery^26^. The methodology and dosage should, therefore, be studied in more detail in the future.

There are some limitations to this study. First, the sample size was small and the results were possibly biased as a consequence. Second, the primary outcome is a calculation based on several clinical measurements that could be a source of error. The hemoglobin measurements can be affected by rehydration in the course of hospitalization and surgery. The type and volume of fluid transfused in each patient was not recorded systematically. Third, we did not record the use of other drugs (e.g. painkillers) in the population. Fourth, the long‐term impact on the incidence of thrombosis was not determined. These are possible confounders that were not accounted for in the study.

### *Conclusions*

This single‐blind randomized controlled trial suggests that the use of TXA in intertrochanteric fracture surgery significantly reduces the risk of intraoperative, hidden, and total blood loss in addition to the need for allogeneic transfusion, without increasing other complications, especially that of DVT and PE. However, larger high‐quality prospective trials are required to strengthen these conclusions, define an optimal regimen, and assess the safety and cost‐effectiveness of TXA before its use can be recommended in intertrochanteric fracture surgery.

## Disclosure

The authors declare that they have no conflict of interest.
